# Dynamic risk stratification of worsening heart failure using a deep learning-enabled implanted ambulatory single-lead electrocardiogram

**DOI:** 10.1093/ehjdh/ztae035

**Published:** 2024-05-08

**Authors:** James Philip Howard, Neethu Vasudevan, Shantanu Sarkar, Sean Landman, Jodi Koehler, Daniel Keene

**Affiliations:** National Heart and Lung Institute, Imperial College London, Du Cane Road, W12 0HS, London, UK; Research and Technology, Cardiac Rhythm Management, Medtronic Inc., Minneapolis, MN, USA; Research and Technology, Cardiac Rhythm Management, Medtronic Inc., Minneapolis, MN, USA; Research and Technology, Cardiac Rhythm Management, Medtronic Inc., Minneapolis, MN, USA; Research and Technology, Cardiac Rhythm Management, Medtronic Inc., Minneapolis, MN, USA; National Heart and Lung Institute, Imperial College London, Du Cane Road, W12 0HS, London, UK

**Keywords:** Ejection fraction, Heart failure, Neural network, Deep learning, Electrocardiogram

## Abstract

**Aims:**

Implantable loop recorders (ILRs) provide continuous single-lead ambulatory electrocardiogram (aECG) monitoring. Whether these aECGs could be used to identify worsening heart failure (HF) is unknown.

**Methods and results:**

We linked ILR aECG from Medtronic device database to the left ventricular ejection fraction (LVEF) measurements in Optum^®^ de-identified electronic health record dataset. We trained an artificial intelligence (AI) algorithm [aECG-convolutional neural network (CNN)] on a dataset of 35 741 aECGs from 2247 patients to identify LVEF ≤ 40% and assessed its performance using the area under the receiver operating characteristic curve. Ambulatory electrocardiogram-CNN was then used to identify patients with increasing risk of HF hospitalization in a real-world cohort of 909 patients with prior HF diagnosis. This dataset provided 12 467 follow-up monthly evaluations, with 201 HF hospitalizations. For every month, time-series features from these predictions were used to categorize patients into high- and low-risk groups and predict HF hospitalization in the next month. The risk of HF hospitalization in the next 30 days was significantly higher in the cohort that aECG-CNN identified as high risk [hazard ratio (HR) 1.89; 95% confidence interval (CI) 1.28–2.79; *P* = 0.001] compared with low risk, even after adjusting patient demographics (HR 1.88; 95% CI 1.27–2.79 *P* = 0.002).

**Conclusion:**

An AI algorithm trained to detect LVEF ≤40% using ILR aECGs can also readily identify patients at increased risk of HF hospitalizations by monitoring changes in the probability of HF over 30 days.

## Graphical Abstract

**Graphical Abstract:**
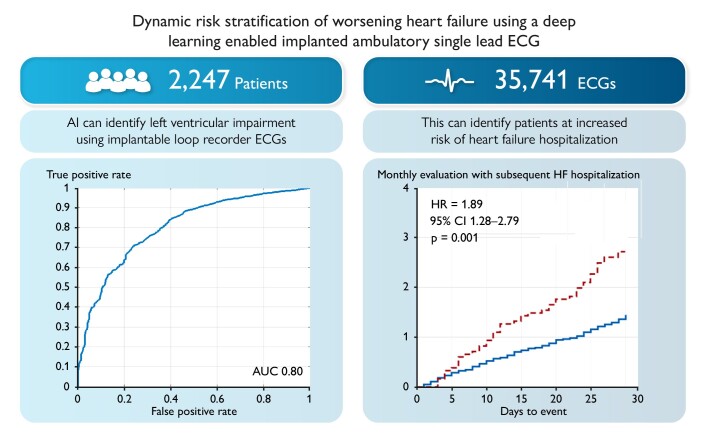

**See the editorial comment for this article ‘Implantable cardiac monitors: the digital future of risk prediction?’, by A. Bauer and C. Dlaska, https://doi.org/10.1093/ehjdh/ztae036.**

## Introduction

Heart failure (HF) is a chronic disease with high prevalence, morbidity, and mortality. The impact of HF is expected to increase substantially with the aging of the population.^1^ The overall economic cost of HF in 2012 was estimated at $108 billion per annum based on a study of patient population across the globe.^2^ With an aging, rapidly expanding and industrializing global population this value will continue to rise. Considering the increasing economic burden of HF, early identification of patients at increased risk of clinical deterioration and hospitalization even prior to the development of severe symptoms may provide an opportunity for early intervention that can prevent worsening of symptoms, slow down disease progression, and improve patient outcomes.^3–10^

Electrocardiogram (ECG) is a commonly adopted non-invasive method for screening and diagnosing cardiovascular disease, and the use of standard 12-lead ECG for systolic HF diagnosis has been ongoing since 1989, advancing from identification of simple abnormalities on ECG to more advanced artificial intelligence (AI) algorithms.^11–16^ These algorithms can predict the presence but also development of HF with more accuracy than traditional ECG-derived metrics. However, no system exists which provides continuous 12-lead ECG monitoring in outpatients. Conversely, over a million implantable loop recorders (ILRs) have been implanted and provide continuous single-lead ambulatory electrocardiograms (aECGs).

Although ILR helps to diagnose arrhythmias, whether this single-lead ECG can be used to predict systolic HF events is unknown.

In this study, we show that AI can analyse aECGs from ILRs to identify patients with reduced left ventricular ejection fraction (LVEF) (≤40%). Furthermore, we show by continuously monitoring daily aECGs for changes in adverse features, we can stratify patients into low- and high-risk cohorts.

## Methods

### Cohort and study design

We conducted a retrospective study of patients in the Optum^®^ de-identified electronic health record (EHR) dataset between 2007 and 2021 from multiple hospital systems in the USA. Through a methodology compliant with health insurance portability and accountability act (HIPAA)’s de-identification standard, a third party determined which of those patients with LVEF measurements from the Optum^®^ EHR were concomitantly enrolled in the Medtronic DiscoveryLink data warehouse. This is a manufacturer’s de-identified device data warehouse containing continuous ECG data from fully ILRs. For patients whose data appeared in both datasets, a combined dataset was created that met HIPAA’s de-identification standard. This process is discussed in detail in the Supplementary Appendix. The combined dataset was used to develop the aECG-convolutional neural network (CNN) algorithm. This retrospective analysis using de-identified data falls into the category of non-human research, and no institutional review board approval was indicated.

### Development of an ambulatory electrocardiogram-convolutional neural network model to detect left ventricular ejection fraction ≤ 40%

**Figure 1:**
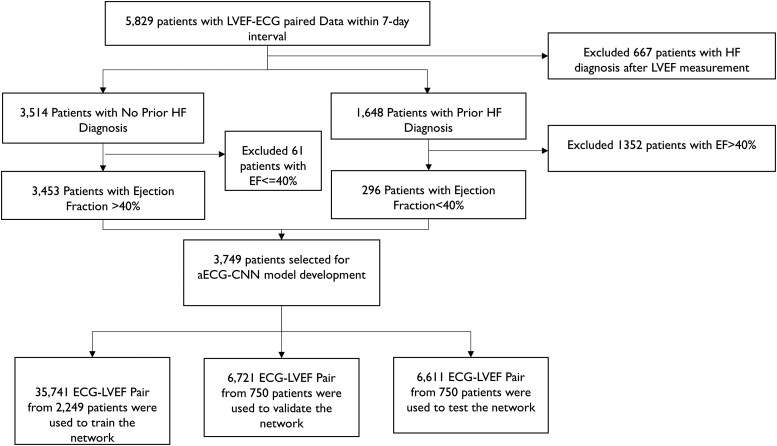
Dataset creation for the ambulatory electrocardiogram-convolutional neural network model development. Schematic indicates the strategy to obtain robust and reliable dataset for model development. To avoid cross-contamination, no patient data are repeated among training, validation, and testing datasets. *Patient count (*n* = 5829) selected based on several inclusion, exclusion criteria to ensure the quality of LVEF data as they are obtained from Optum^®^ EHR and the dataset was captured in Optum^®^ EHR via natural language processing of procedure/diagnostic notes and prone to natural language processing errors.

To create the aECG-CNN model development dataset, LVEF measurements from Optum^®^ EHR were matched to single-lead aECGs measured within 7 days (before/after) of the LVEF measurement date. We excluded patients diagnosed with hypertrophic cardiomyopathy (ICD10: I42.1, I42.2; ICD9: 42511, 42518). To ensure robust and reliable LVEF information and minimize errors introduced through automated data extraction from Optum^®^ EHR (*Figure 1*), we only included patients with prior HF diagnosis to create the low ejection fraction (EF) data (LVEF ≤ 40%). Patients with no HF diagnosis and an EF >40% were used to create the normal EF data (LVEF > 40%). More detailed information on data cleanup is provided in the supplementary material (see Supplementary material online, *Figure S1*). Electrocardiograms were randomized at the patient level to the training, validation, and testing datasets using a 60%:20%:20% split.

A custom deep learning convolutional neural network model (aECG-CNN) was trained on an independent training database of LVEF-aECG pairs to detect LVEF ≤ 40% (full architecture listed in the Supplementary Appendix). The model has 24 layers with 1.7 M total learnable parameters. The algorithm predicts the probability (0–1) of LVEF ≤40% from 10 s of aECG data; one indicates the highest probability of LVEF ≤40%. Cross-entropy loss was calculated and minimized using the adaptive moment estimation optimizer.^17,18^ During training, we performed data augmentation through over-sampling of low EF ECGs, Gaussian noise, and cyclical shifting of the signal. There was no test-time augmentation.

### Longitudinal analysis of ambulatory electrocardiogram and assessment of increased risk of future heart failure hospitalization events

**Figure 2:**
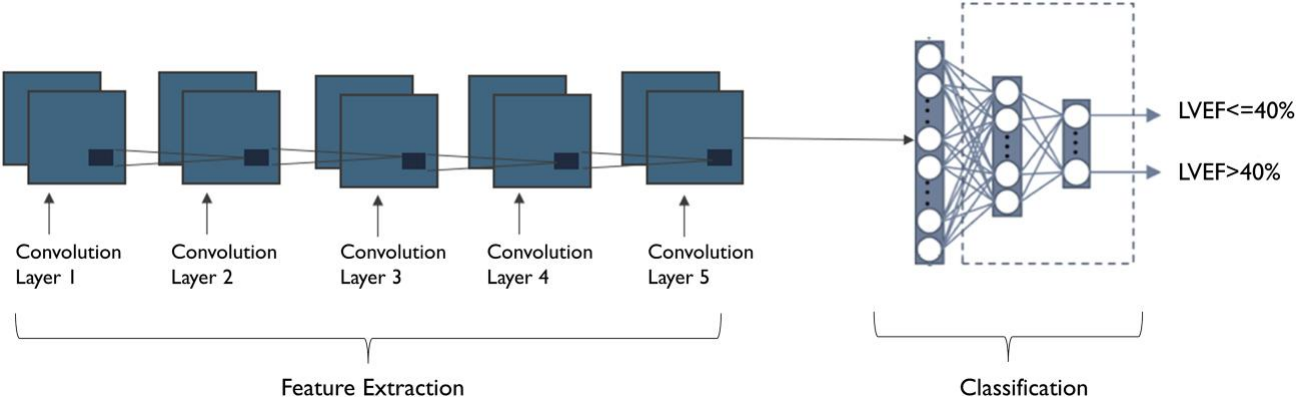
Ambulatory electrocardiogram-convolutional neural network model architecture to detect low ejection fraction. Input to the model is scalograms (image) of single-lead ambulatory electrocardiogram signal.

There is evidence that fluctuations in LVEF can predict HF hospitalizations.^19,20^ We hypothesized that the aECG-CNN algorithm has the potential to track the changes in probabilities of LVEF ≤40% using longitudinal aECG data and the degree of change/relative change can be used to assess the dynamic risk of future HF hospitalization events (*Figure 2*).

The proposed algorithm uses the fact that ILRs record a daily ECG and that changes in LVEF may occur over both shorter and longer periods. Therefore, we calculate three running averages of the low EF score (using the ECG) over short (7-day) and medium (15-day) intervals and compare these to a 30-day baseline for each patient. These periods were chosen empirically to reduce the risk of overfitting to the dataset; it was hypothesized that shorter periods than 7 days would comprise too few measurements and periods longer than 15 days risked ‘missing’ the change to act. The specific rules used to assign patients to the high- and low-risk cohort are shown in *Table 1*.

**Table 1:** Dynamic risk group stratification based on aECG-CNN probability changes within a month

Risk assessment	Feature set
High risk	Defined as **large fluctuations** and **sustained fluctuations** in predicted LVEF.
	**Large fluctuations** = (Pmax7-Pmax30 ≥ 0.1) AND (Pmax15-Pmean15 ≥ 0.05)
	**Sustained fluctuations** = (Pmax7-Pmax15) ≥ 0.08) for ≥6 Days OR (Pmax7-Pmax30) for ≥5 Days
Low risk	Above conditions not met.
Utilized features	Description
Pmax7	The peak probability of LVEF ≤ 40% within the 7-day running average.
Pmax15	The peak probability of LVEF ≤ 40% within the 15-day running average.
Pmean15	The mean probability of LVEF ≤ 40% within the 15-day running average.
Pmax30	The peak probability of LVEF ≤ 40% within the 30-day running average.

**Figure 3:**
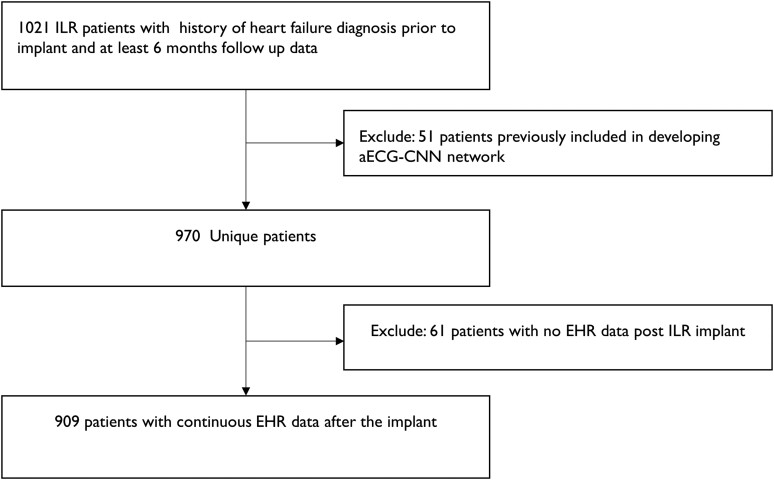
Schematic indicates the dataset selection for longitudinal analysis of ambulatory electrocardiogram-convolutional neural network algorithm to assess the increased risk of future heart failure events.

To test this algorithm, we used a real-world cohort of ILR patients with HF hospitalization prior to implant and follow-up ECGs for at least 6 months post-implant (*Figure 3*). Heart failure hospitalization was used as the endpoint and was defined as an inpatient, emergency department, or observation unit stay in a hospital with a primary discharge diagnosis of HF. Primary diagnosis of HF was ascertained based on ICD9/ICD10 codes: 428.X, 402.01, 402.11, 402.91, 404.01, 404.03, 404.11, 404.13, 404.91, 404.93, I50.X, I11.0, I13.0, and I13.2 as has been described previously.^21^

**Figure 4:**
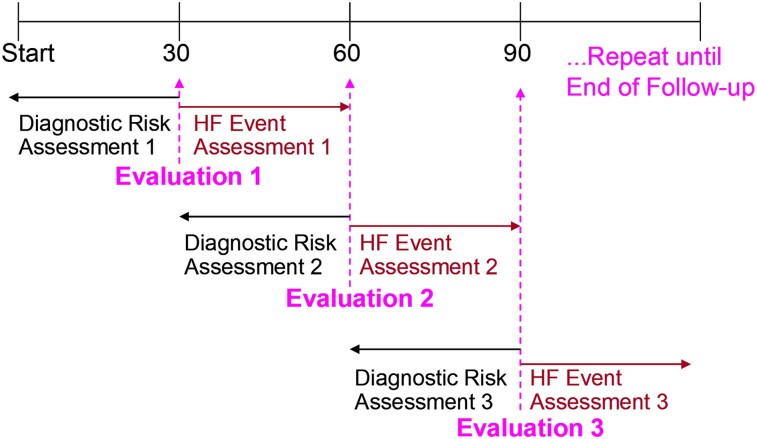
Every 30 days the previous 30 days are evaluated for risk group based on ambulatory electrocardiogram-convolutional neural network, and then, the subsequent 30 days are evaluated for heart failure event. Start indicates implant date.

The ability of aECG-CNN to predict HF hospitalization was assessed by simulating monthly follow-ups which consisted of looking at the aECG-CNN risk states across 30 days and evaluating the occurrence of clinical events in the following 30 days (*Figure 4*) as has been described previously.^5^

### Statistical analysis

The performance of the algorithm to identify LVEF ≤40% was assessed using the area under the receiver operating characteristic (AUROC) curve. To assess the algorithm’s ability to predict HF hospitalization, a marginal Cox proportional hazards model was used to calculate the hazard ratios (HRs) and 95% confidence intervals (CIs) for patients identified by the algorithm as high vs. low risk. The marginal model adjusts for multiple observations within individual subjects. Kaplan–Meier analysis was performed. Patients were right censored at the last day of EHR data availability if <30 days. Cox regression models were used for adjusting several clinical variables, including age, gender, race, and comorbidities such as diabetes, hypertension, and history of myocardial infarction (MI). Baseline variables that may be manipulated because of worsening HF (e.g. HF medications) were not adjusted for. All statistical analyses were performed in Minitab (v20.1.3) and SAS (v9.4).

## Results

### Ambulatory electrocardiogram-convolutional neural network algorithm to predict left ventricular ejection fraction ≤ 40%

**Figure 5:**
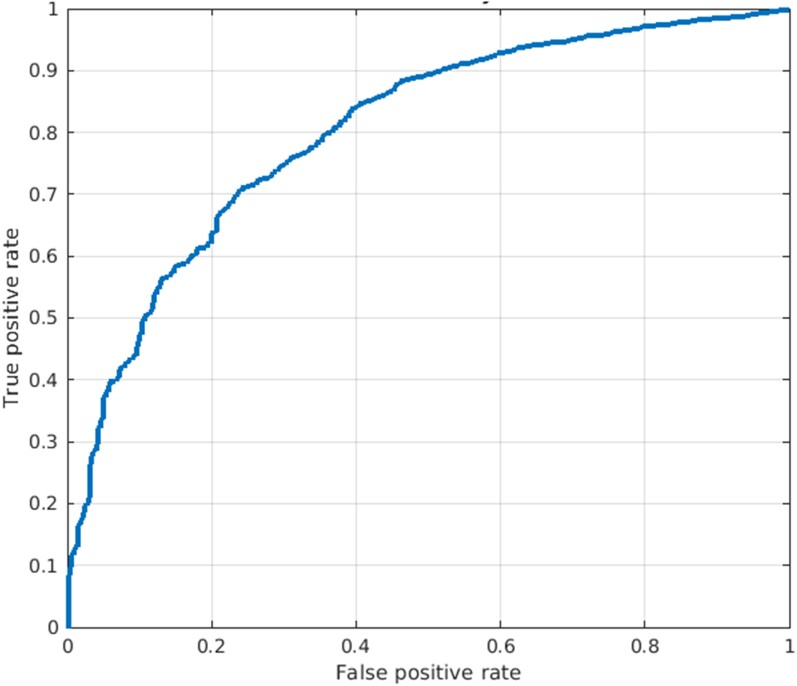
The diagram shows the receiver operating characteristic of ambulatory electrocardiogram-convolutional neural network classification model on test dataset.

The aECG-CNN model was trained on a dataset of 35 741 LVEF-ECG pairs from 2249 patients to predict the probability of LVEF ≤40%. The model was validated on an independent dataset of 6721 LVEF-ECG pairs from 750 patients and tested on another independent dataset of 6611 LVEF-ECG pairs from 750 patients. The baseline characteristics of total dataset used for the development, validation, and testing of the aECG-CNN model are provided in *Table 2*. The model yielded an accuracy, a sensitivity, a specificity, and an AUROC of 75%, 70%, 76%, and 0.8, respectively, in the independent test dataset. Full event counts as a 2 × 2 table, precision, and F1 scores are provided in the Supplementary Appendix, Supplementary material online, *Tables S1* and *S2*. The receiver operating characteristics curve in *Figure 5* provides the performance of model on test dataset.

**Table 2:** Baseline patient characteristics of aECG-CNN algorithm development/validation/testing set

Clinical history	All (*n* = 3749)	Training set (*n* = 2249)	Validation set (*n* = 750)	Test set (*n* = 750)	*P*-value
Mean age (years)	68 (14)	68 (14)	68 (14)	68 (14)	0.69
Male gender	2009 (53.6)	1202 (53.4)	401 (53.4)	406 (54.1)	0.94
Mean LVEF	57.9 (8.8)	57.9 (8.8)	58.1 (8.9)	57.97 (8.9)	0.17
Race					0.43
African American	487 (13.0)	294 (13.1)	110 (14.7)	83 (11.1)	
Caucasian	3006 (80.2)	1794 (79.7)	595 (79.3)	617 (82.2)	
Asian	42 (1.1)	25 (1.1)	9 (1.2)	8 (1.1)	
Others	214 (5.7)	136 (6.1)	36 (4.8)	42 (5.6)	
Ethnicity					
Hispanic	169 (4.7)	105 (4.7)	31 (4.1)	33 (4.4)	0.92
Comorbidities					
Hypertension	2678 (71.4)	1602 (71.2)	535 (71.3)	541 (72.1)	0.89
History of MI	684 (18.2)	405 (18.0)	136 (18.1)	143 (19.1)	0.81
Diabetes mellitus	1133 (30.2)	659 (29.3)	242 (32.3)	232 (30.9)	0.27
Cardiomyopathy	566 (15.1)	336 (14.9)	122 (16.2)	108 (14.4)	0.57
Atrial fibrillation	1154 (30.8)	702 (31.2)	230 (30.7)	222 (29.6)	0.71
Chronic obstructive pulmonary disease	49 (1.3)	33 (1.5)	7 (0.93)	9 (1.2)	0.52
Heart failure	296 (7.9)	177 (7.8)	63 (8.4)	56 (7.5)	0.79

Discrete data are presented as counts (percentage) and continuous measurements as mean (standard deviation).

### Ambulatory electrocardiogram-convolutional neural network algorithm to assess dynamic risk of heart failure hospitalization

**Figure 6:**
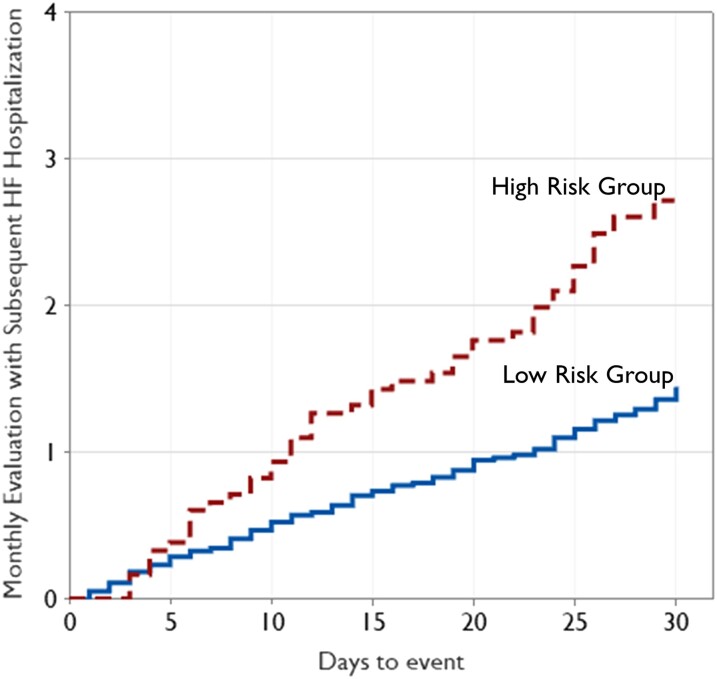
The diagram shows the Kaplan–Meier analysis of cumulative incidence of monthly evaluations with subsequent heart failure hospitalization among high-risk and low-risk category. 494 patients contributed solely to the high-risk group, 901 solely to the low-risk group, and 486 to both groups.

A total of 909 patients with 12 467 follow-up monthly evaluations were analysed to assess the risk of future HF hospitalization based on the relative change in probability of aECG-CNN model inference output. The mean follow-up was 13.7 months from implant date. A total of 201 monthly evaluations (1.6%) had HF hospitalization in the next 30 days. The baseline characteristics of the patient cohort are provided in *Table 3*, including the reason for ILR implantation. The average age of patients was 68 ± 13 years, with 51% males. The prevalence of comorbidities diagnosed prior to ILR implant was as follows: diabetes (39%), hypertension (95%), dilated cardiomyopathy (25%), congestive HF (99%), valvular disease (3%), coronary artery disease (75%), history of MI (56%), renal dysfunction (52%), ventricular arrhythmia (30%), and atrial fibrillation (57%). Based on the change in probability of LVEF ≤40% during monthly evaluations, 1830 evaluations (14.7%) were categorized as high risk and 10 637 evaluations (85.3%) as low risk. *Figure 6* shows the Kaplan–Meier curve of cumulative incidence of monthly evaluations with subsequent HF hospitalization events in the next 30 days among two risk groups identified by aECG-CNN model. Patients aECG-CNN identified as high risk were almost twice as likely to have HF hospitalization (HR 1.89; 95% CI 1.28–2.79; *P* = 0.001) than those it identified as low risk (*Table 4*). This performance was unchanged after adjusting for patient demographics (HR 1.88, 95% CI 1.27–2.79; *P* = 0.002).

**Table 3:** Baseline demographics of patients in the study

Clinical history	Total (*n* = 909)
Mean age	68 (13)
Male gender	464 (51.1)
**Race**	
African American	163 (17.9)
Asian	7 (0.8)
Caucasian	695 (76.5)
Other	44 (4.8)
Ethnicity, Hispanic	44 (4.8)
Baseline LVEF	53.5 (13.5)
NT-proBNP	1512 (3983)
**Comorbidities**	
Diabetes mellitus	354 (38.9)
Hypertension	861 (94.7)
Renal dysfunction	475 (52.3)
History of MI	512 (56.3)
Atrial fibrillation	522 (57.4)
Ventricular arrhythmias	277 (30.5)
Coronary artery disease	684 (75.2)
Congestive heart failure	899 (98.9)
Dilated cardiomyopathy	225 (24.7)
Hypertrophic cardiomyopathy	16 (1.7)
Valvular heart disease	28 (3.1)
**Medications history**	
Angiotensin-converting enzyme inhibitors/Angiotensin receptor blockers	478 (52.6)
Anticoagulants	494 (54.3)
Diuretics	811 (89.2)
Entresto	21 (2.3)
Spironolactone	219 (24.1)
Beta-blockers	710 (78.1)
Vasodilator–nitrate	468 (51.5)
**Antiarrhythmic Drugs**	
Class I/III/IV	289 (31.8)
Class I	48 (5.2)
Class III/IV	272 (29.9)
**Reason for ICM monitoring**	
AF ablation monitoring	33 (3.6)
AF management	126 (13.8)
Cryptogenic stroke	162 (17.8)
Palpitations	42 (4.6)
Suspected AF	62 (6.8)
Syncope	341 (37.5)
Ventricular tachycardia	21 (2.3)
Other/unknown	122 (13.4)

Discrete data are presented as counts (percentage) and continuous measurements as mean (standard deviation).

**Table 4:** HF event rate comparison between different risk groups based on aECG-CNN in ILR patients with history of HF events any time prior to implant

Diagnostic parameter	Number of evaluations (%)	Number of HF events (% of evaluations)	Hazard ratio (95% CI)	*P*-value
**Risk group based on aECG-CNN**				0.001
Low	10 637 (85.3)	152 (1.4)	Reference	
High	1830 (14.7)	49 (2.7)	1.89 (1.28–2.79)	

## Discussion

In this study, we show that an AI algorithm trained to identify patients with impaired LVEF from ILR aECGs can be used to dynamically predict the risk of HF hospitalization over the next 30 days. Early identification of these patients would allow timely cardiovascular care including appropriate diagnostic testing, medication optimization, or even device-based therapies if deemed necessary which may improve patient outcomes and ease acute healthcare provision resource utilization.^3,6,8^

This model has the potential to reside in a remote monitoring platform and continuously access the changes in aECG to monitor the probability of an instance of LVEF ≤ 40% and subsequent increase of worsening HF hospitalization risk. Previous studies have shown the utility of standard 12-lead ECG markers to assess future HF events, and indeed, some of these report higher area under the curves (AUCs) than this study.^11–15,22–25^ However, these previous studies typically rely on 12-lead ECGs. This requirement limits the clinical utility of this approach. No technology exists that readily permits the regular automated gathering of continuous 12-lead aECGs that could serve as a telemonitoring solution. Ambulatory electrocardiogram-CNN, however, provides a daily single-lead aECGs from ILRs to provide the required continuous dynamic daily risk score for HF hospitalization, at no extra burden for patients with these devices. Such a system may be analogous to other implantable monitoring techniques that exist for ambulatory monitoring of increased risk of worsening HF.^3–10^ However, by using the ECG to directly predict cardiac function, this approach may provide more orthogonal inputs to existing biosensors of pressure, impedance, and physical activity.

Besides providing ability for diagnosis and monitoring of cardiac arrhythmia,^26–29^ the ILR devices also monitor diagnostic parameter and store aggregated daily measurements longitudinally over a long period of time. These diagnostic parameters include nighttime heart rate and daytime heart rate, atrial fibrillation burden, ventricular rate during atrial fibrillation (AF), heart rate variability, and activity duration. Additionally, other sensors such as subcutaneous impedance are being investigated for measurement of fluid and respiration rate. Recently, an approach of combining multiple diagnostic parameters in an ILR device using a Bayesian belief network machine learning model for identifying patients at risk of worsening HF was reported for ILR devices in patients with HF with reduced and preserved EF.^30^ Ambulatory electrocardiogram-CNN could readily be added into these ambulatory HF event risk prediction platforms potentially delivering further improvement in the performance of the currently available multi-parameter algorithms. Whether such a risk scoring methodology for ambulatory management of patients with HF will improve patient outcomes requires prospective evaluation. A randomized control study is currently being conducted to investigate whether ILR-based ambulatory management can improve outcomes in Class II and III patients with HF with reduced LVEF or preserved LVEF (ALLEVIATE-HF study: NCT04452149).

Finally, the aECG-CNN-based monitoring can also identify patients with potential asymptomatic or mildly symptomatic reduction in LVEF in an ambulatory setting—indeed, this is what the model was trained to identify. If the model were to suspect new left ventricular impairment in a previously healthy patient, this could trigger confirmatory diagnostic testing (e.g. echocardiography) which could lead to the earlier identification of patients who could benefit from prognostic medication and device therapy again with potential benefits for patients and healthcare services alike.

### Study limitations

We have relied on EHRs for identifying LVEF measurements, clinical endpoints, and the presence of comorbidities. Such approaches are susceptible to data entry errors and faults in natural language processing. We aimed to minimize these by requiring concordant ICD codes.

Single-lead aECG is derived from a short dipole, in a non-standard orientation, which varies from patient to patient. This almost certainly limits the ability of aECG-CNN to identify pathology and predict events when compared with standard 12-lead ECGs. However, because ILRs provide continuous daily ECG monitoring, our system can rely on intra-patient changes in HF probabilities, which is not currently available with 12-lead ECGs.

Although this study included over 2000 patients, a prospective study could be useful in demonstrating the system’s clinical utility. First, this would demonstrate the generalizability of the algorithm to an external prospective dataset, rather than an anonymized electronic health record. Second, this would allow the additive predictive power of the system to be ascertained, beyond those of existing telemonitoring biosignals.

## Conclusions

This study found that an aECG-CNN algorithm trained to detect reduced LVEF using single-lead aECGs acquired by an ILR can identify patients at increased risk of future HF events. Further investigation is needed to evaluate whether HF risk prediction based on this ECG-based deep learning model can be used to predict and prevent HF events in a prospective study. If this is found to be the case, the benefits for patients and healthcare systems alike would likely be large.

## Supplementary Material

ztae035_Supplementary_Data

## Data Availability

All patients provided consent to use their de-identiﬁed device data for research purposes when they sign up for Medtronic CareLink Network. As per the contractual data access, the de-identified data cannot be shared.
