# Supplementary material for: Dynamic risk stratification of worsening heart failure using a deep learning-enabled implanted ambulatory single-lead electrocardiogram
Source: Eur Heart J Digit Health. 2024 May 8;5(4):435–43. doi: 10.1093/ehjdh/ztae035 (PMC11284004; doi:10.1093/ehjdh/ztae035)
Supplement: ztae035_Supplementary_Data [file ztae035_supplementary_data.docx]

Supplementary Appendix

# Anonymisation and Linking process


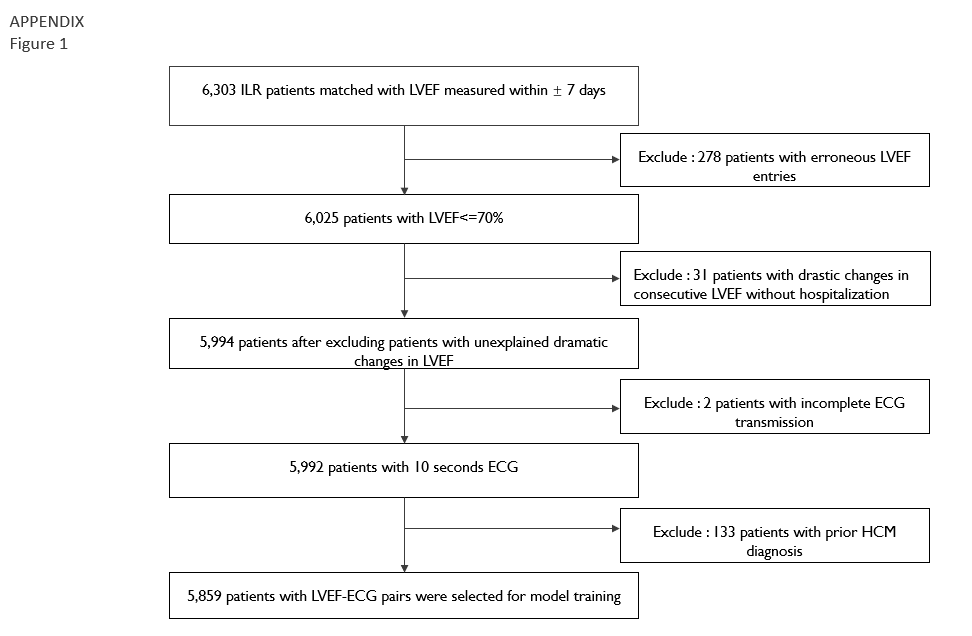


**Appendix Figure 1.** Schematic of data cleanup process to obtain robust and reliable LVEF information from Optum® EHR dataset.

The patient matching process involved the following processes to ensure HIPAA compliance:

**1. Patient Matching**

Patients from Medtronic’s Carelink warehouse are first matched to Optum’s EHR database using seven identifiers. These identifiers are hashed (encrypted) by each company on their respective premises and then securely uploaded to a third-party vendor who runs an overlap report of the matched patients.

**2. Risk Determination**

After patient matching, Medtronic collects device information for the matched patients; Optum does likewise for the EHR information of matched patients. Before linking the two datasets together, a third-party statistical expert evaluates the data dictionary for each dataset as well as the combined data dictionary. Samples of data are also collected from each respective dataset to determine frequencies and to calculate re-identification risk thresholds.

**3. Data Remediation**

Where the statistical expert identifies a risk of re-identification in the combined dataset, data from either Medtronic or Optum or both is removed or aggregated and then re-submitted for re-evaluation. The process repeats until the expert determines that the combined dataset has a low risk of re-identification.

**4. Certification**

A letter of determination, or certification, is issued before the two datasets are combined.

**5. Rekeying of Identifiers**

After certification, Optum combines both datasets and rekeys all internal identifiers. This includes fields like patient, provider, device, and episode identifiers. The result is a final dataset that cannot be joined to previous data deliveries or internal data sources.

**6. Governance & Training**

The dataset is stored on its own Medtronic platform with restricted access. Users cannot connect the data between systems and receive training on how to report summary results without revealing low counts.

# Input data and neural network architecture

In the preprocessing step, we convert raw ECG waveforms to 108 different time-frequency components.

The time frequency data are stored as two-dimensional arrays of 224 x 224 pixels, allowing the use of a 2 dimensional convolutional neural network. This network comprised 5 convolution layers using an increasing number of filters as the network became deeper (32, 64, 64, 96, 96). The first layer was followed by a MaxPooling layer, and all subsequent layers were followed by an AveragePooling layer. ReLU was used as the activation function throughout. The filter dimension was 5 x 5 (as opposed to the more typical 3 x 3 filters). This results in a total number of learnable parameters of 1.7M.


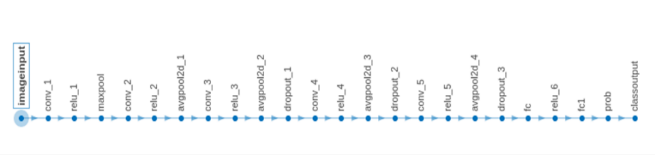


# Marginal Means and Performance Metrics for identifying low left ventricular ejection fraction

## Confusion Matrix with Counts and Margins

|  | Predicted Positive | Predicted Negative | Total |
| --- | --- | --- | --- |
| Actual Positive | 321 | 140 | 461 |
| Actual Negative | 1482 | 4668 | 6150 |
| Total | 1803 | 4808 | 6611 |

## Performance Metrics

| Metric | Value |
| --- | --- |
| Accuracy | 75% |
| Sensitivity | 70% |
| Specificity | 76% |
| AUC | 0.8 |
| Precision | 18% |
| F1 | 28% |
